# Isolated Multiple Pigment Epithelial Detachments with Unknown Cause

**DOI:** 10.1155/2014/289107

**Published:** 2014-02-04

**Authors:** Arzu Seyhan Karatepe Hashas, Altan Göktas, Mustafa Atas

**Affiliations:** Department of Ophthalmology and Vision Sciences, Kayseri Training and Research Hospital, 38010 Kayseri, Turkey

## Abstract

There are many etiological factors that have led to the development of retinal pigment epithelial detachment (PED). In this paper, we have reported a patient with isolated multiple PEDs. Based on this fact, this paper aimed to give an overview of the causes of PEDs.

## 1. Introduction

Retinal pigment epithelial detachment (PED) results in the separation between the retinal pigment epithelium (RPE) basement membrane and the inner collagenous layer of the Bruch's membrane [1, 2].

It is not really an illness; however, it is an ocular finding which can be observed in several chorioretinal diseases such as central serous chorioretinopathy (CSC), age-related macular degeneration (AMD), and several inflammatory and ischemic chorioretinal diseases [3].

Several hypotheses have been proposed for the formation of pigment epithelium detachment (PED). In the past, PED was interpreted as a kind of fluid leakage from increased intravascular pressure of choroidal system [4].

The prevailing opinion was that, because of the debris collected in the inner layers of Bruch's membrane, the physical relationship between Bruch's membrane and the RPE may weaken, and thus choroidal fluid may passively accumulate under the retinal pigment epithelium (RPE).

The filling pattern shown by fluorescein angiography (FA) and the impaired Bruch's membrane structure supported this opinion [4].

However, Gass argued for a second opinion suggesting that PED may occur as a result of fluid leakage from the neovascular vessels proceeding in the inner layers of Bruch's membrane, and this fluid may be an obstacle to display these vessels in angiography.

According to the current hypothesis, the source of the fluid in PED, contrary to the prevailing opinion until the year 1986, can be the defect which occurred in elimination metabolism of RPE rather than choroid. It is hypothesized that the decrease in the hydraulic conductivity of Bruch's membrane towards choroid can lead to accumulation of fluid in the subpigment epithelial field. At this stage, the hydrophobic character of Bruch's membrane causes the development of resistance against fluid flow. This pathogenetic explanation for RPE detachment is found more challenging than previous concepts [4].

In this case, as a result of thickening of Bruch's membrane and the hydrophobic character, active metabolic wastes, which originated from pigment cells, fail to reach choroidal circulation, and thus secondary choroidal neovascularization occurs.

Today, in addition to these mechanisms, together with increased levels of “vascular endothelial growth factors,” choroidal neovascularization and fluid leakages from already existing neovascularization are known to increase, as well. Through inhibiting increased levels of VEGF, management of fluid withdrawal and reduction of neovascularization are intended in the treatment of PED in age-related macular degeneration (AMD) [5].

Although none of these mechanisms could explain the mechanism of PED alone, on the ground set by certain changes due to aging, as well as genetic and environmental predispositions, PED develops with the contribution of these mechanisms. In the clinically diagnosed PED patients, diagnosis can be confirmed with optical coherence tomography (OCT), which is an easy and noninvasive method to examine subpigment epithelial fluid and concomitant drusen, intraretinal fluid, and other findings. OCT may be clinically used to monitor the course of treatment. Fundus fluorescein angiography (FA) is another important method for diagnosis and differential diagnosis of PED.

In this paper, we have presented the clinical features of a patient with bilateral isolated PED and probable leading causes are discussed.

## 2. Case 

**Figure 1:**
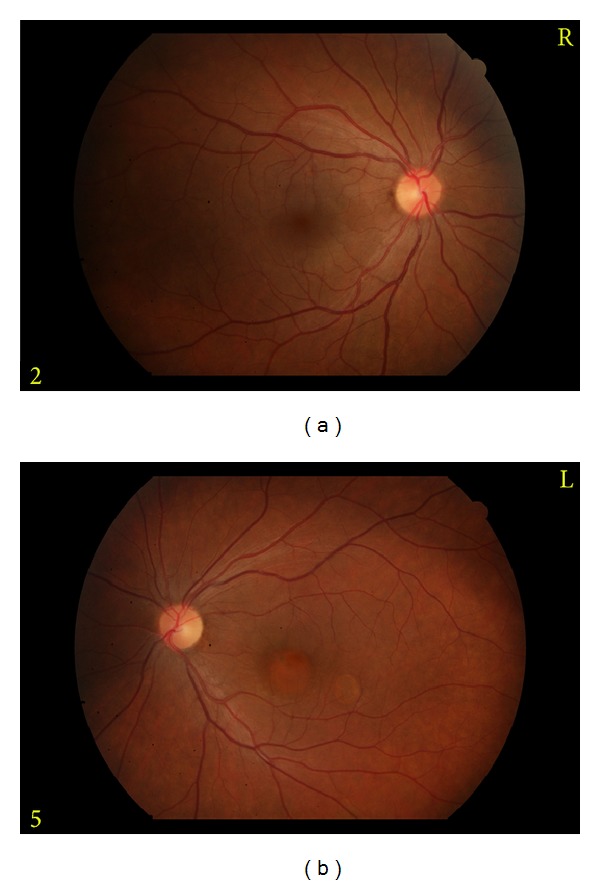
A small vesicle under the superior temporal quadrant of the right eye (a) and two separate vesicles filled with liquid in the left eye (b).

**Figure 2:**
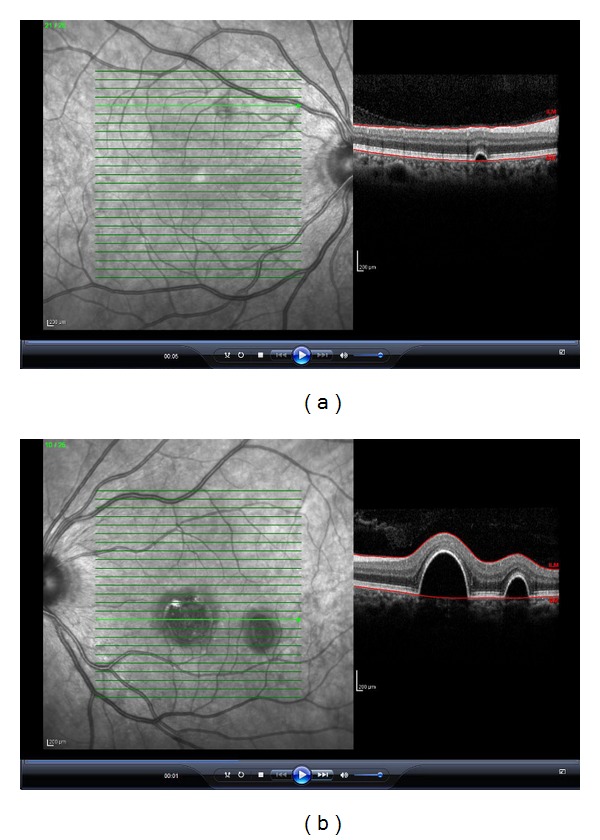
OCT cross-section images of these lesions showed serous PED in the right eye (a) and in the left eye (b).

**Figure 3:**
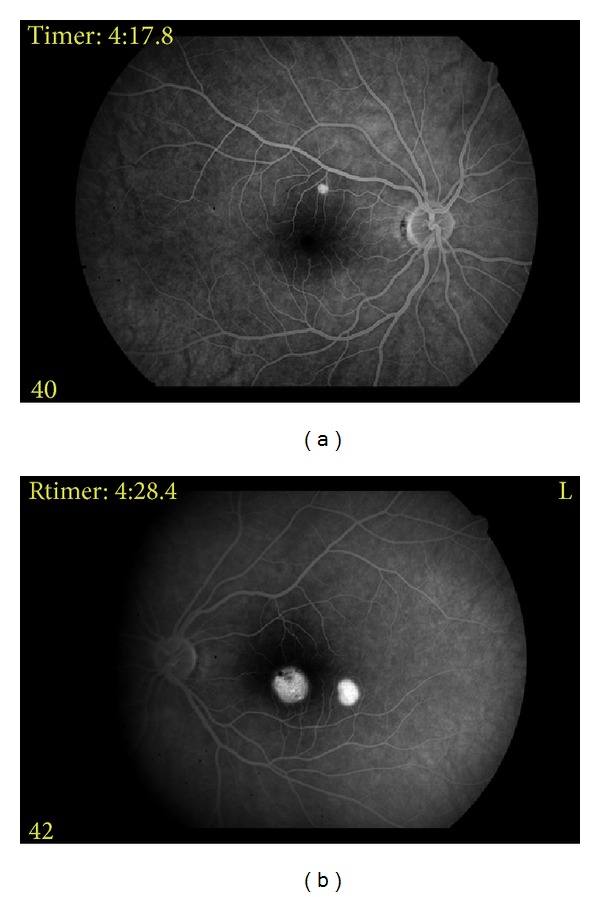
Early-phase FA revealed hyperfluorescent areas (fluorescence pooling) in PED but no findings such as leakage or fluorescence expansion could be detected ((a), (b)).

27-year-old female patient referred to our clinic with the complaint of decreased vision in the left eye. She stated that her complaint lasted for the last 6 months, but she did not apply to any doctor in this period. No other remarkable characteristics were noted in her history. In the examination, best-corrected visual acuity (VA) was 0.9 in the right eye, while 0.3 in the left eye. Intraocular pressure (IOP) and anterior segment examination yielded normal results, and dilated fundus examination was performed. Fundus examination revealed a small vesicle under the superior temporal quadrant of the right eye, whereas two separate vesicles filled with liquid in the left eye, one in the size of the optic disc at the center of fovea and other smaller one at the lower temporally of fovea (Figure 1). OCT cross-section images of these lesions showed serous PED (Figure 2). No concomitant lesion was determined. Early-phase FA revealed hyperfluorscent areas (fluorescence pooling) in PED but no findings such as leakage, or fluorescence expansion were detected (Figure 3).

## 3. Discussion 

Different PED classifications based on clinical and angiographic appearances are available. Poliner et al. [6] classified PED as serous, turbid, and hemorrhagic in 3 groups.

Casswell et al. [7] classified PED into 5 groups according to fluorescein angiographic appearances as follows: (I) early hyperfluorescence, (II) late hyperfluorescence, (III) shallow and limited hyperfluorescence, (IV) irregular hyperfluorescence (fibrovascular PED, hyperplastic, or elevated area of RPE field that can block fluorescence, serous detachment of RPE, hemorrhagic detachment of RPE, drusenoid detachment of RPE) and (V) large confluent drusen area.

Hartnett et al. [8] classified PED into six groups: (1) pseudovitelliform PED, (2) confluent drusen type PED, (3) serous PED, (4) vascular PED, (5) hemorrhagic PED, and (6) retinal vascular anomaly PED.

Serous PED is dome-shaped elevations of the RPE, with sharp borders. It can be transparent, turbid, or lipidous according to the contents of the underlying liquid. Definitive diagnosis can be managed with a careful ophthalmoscopic examination. OCT reveals focal areas of RPE with an increased reflectivity over an optically clear space. Based on the morphological changes, the detached RPE is more reflective than normal RPE; increased reflectivity of RPE may significantly shade the choroidal reflectivity, and a narrow-angled detachment edge is detected due to the tight coupling between the RPE and Bruch's membrane [9].

OCT findings of our case comply with typical serous PED. RPE detachment showed higher reflectivity and thus shaded choroidal reflectivity.

Early phases of a serous PED show a uniform hyperfluorescence of the entire PED lesion in FA; the fluorescence of the PED lesion increases as well with the increased fluorescein dye but does not exceed the borders of the lesion; in the late phases PED shows well-demarcated pooling of the dye. In the old PEDs, irregular fluorescence blocking due to pigment migration can be seen [10].

In both eyes of our case, the FA and OCT findings were typical of serous PED. Well then, in which diseases can these serous PEDs typically occur as an indicative symptom?

PED is a common finding in central serous chorioretinopathy (CSC). Patients with CSC, which can be called idiopathic choroidal vascular hyperpermeability, are more likely to have type A personalities [11].

A case-control study determined the use of corticosteroids and have hypertension as an important risk factor for patients with CSC [12].

There are two main types of CSC. “Typical” or “classic” type is usually seen in younger patients. This type causes an acute localized detachment of the retina with mild to moderate loss of visual acuity associated with one or a few focal leaks seen during fluorescein angiography. “Diffuse retinal pigment epitheliopathy,” “decompensated RPE,” or “chronic CSC” has widespread alteration of pigmentation of the RPE in the posterior pole related to the chronic presence of shallow subretinal fluid [13, 14].

In acute CSC cases, neurosensory detachment associated with PED is a common finding in OCT. In chronic CSC cases, loss of photoreceptor outer segments, flattened foveal contours, thinning of the retina, and widespread RPE irregularities can be observed on OCT.

Serous PEDs are reported frequently in conjunction with central serous chorioretinopathy [15]. In addition, some originally serous PEDs have been shown to transform to typical CSC, which include other components as well. Bandello et al. described an uncommon case of a 25-year-old woman affected by bilateral idiopathic multiple serous detachments of the macular retinal pigment epithelium. During the fluorescein angiography follow-up, in either macular area, one of these detachments resulted in a typical central serous chorioretinopathy active leakage point. These findings detail that idiopathic serous detachments of the retinal pigment epithelium may represent predisposing changes for the development of macular neurosensory retinal detachment. However, there is no study available indicating from which PEDs CSC may develop [15, 16].

After photocoagulation treatment of isolated serous PEDs, regression of the lesion is a similar finding with CSC. Curing with the same treatment may suggest a similar pathogenesis for PED and CSC [17].

Giovannini et al. [18] observed that PEDs frequently are associated with choroidal leakage and venous dilatation, and this supports the hypothesis that an idiopathic serous pigment epithelium detachment is a variant of central serous chorioretinopathy.

PED is a nonspecific finding. Other than CSC, AMD is another disease that PED can be detected. AMD occurs more frequently in the elderly; however, symptoms such as drusen, subretinal neovascularization, intraretinal or subretinal fluid, and geographic atrophy generally accompany PED. PEDs detected in AMD are usually fibrovascular PEDs. FA findings, particularly slow filling, delayed filling, irregular filling, and notching, indicate the presence of PED in AMD [10].

Demonstrating the polypoid dilatation areas in the indocyanine green angiography (ICG) in polypoid choroidal vasculopathy (PCV), which is recognized as a subtype of AMD, may be useful for definitive diagnosis. Other findings are similar to that of AMD.

Lumbroso et al. [19] have investigated the relationship between morphological differences and etiology in PEDs by using enhanced deep imaging spectral domain OCT (EDI SD-OCT) examination of 30 eyes of 22 patients and determined that PED shape was circular in 88.8% of the CSC patients, with a smooth inner appearance, while irregular or multilobular in 76.2% of AMD patients with granular inner appearance. Clear PEDs generally accompanied CSC.

Our patient was within the common age range of CSC. Besides the age, the lack of accompanying symptoms such as drusen and choroidal neovascularization in the patient led our diagnosis away from AMD. OCT and FA findings that were compatible with serous PED supported the diagnosis of CSC. As it was pointed out by Lumbroso et al., the circular, smooth, and transparent features that are required for the diagnosis of CSC were totally compatible with the findings of our PED case.

Another disease where PED can be seen is hypertensive choroidopathy. It is diagnosed with high arterial blood pressure, accompanying Elschnig spots, which are focal RPE hyperpigmentation areas surrounded by hypopigmented areas as a result of choroidal ischemia. PED can be observed also in acute retinal pigment epitheliitis (Kyrill disease). It is presented with decreased VA and central scotoma in healthy young adults and thus can be confused with CSC. However, it can be differentiated from CSC with hypopigmented halos surrounding macular lesions and also with the lack of leakage from hyperfluorescent pigment clusters, which are similar to Elsching spots [7].

We did not consider hypertensive choroidopathy and Kyrill disease in our patient because we did not observe concomitant hypertension and pigment changes. Other causes of serous detachment, such as choroidal tumor, choroiditis, retinal vein occlusion, and optic nerve pits, can easily be excluded from isolated PED through a detailed fundus examination, OCT and FA and also because of the concomitant lesions.

In the light of all these data, our patient is considered as an isolated serous PED case. Without the other symptoms of CSC and another eye disease, our case is important to indicate that PEDs can be found by being isolated. Lack of concomitant lesions and OCT findings like multiple, bilateral, serous, well-demarcated, and circular PED lesions, as well as FA findings like hyperfluorescence from the early phases on and lack of leakage, led us to conclude as isolated PED. It is uncertain if other findings may be added and CSC may develop in the future. Monitoring the patient for a long period of time will clarify if this is a pure isolated PED case or the initial stage of CSC.
